# Systematic evaluation of magnetic particle imaging quantification strategies in biologically relevant scenarios using anatomically correct 3D printed mouse phantoms

**DOI:** 10.1088/1361-6560/ae91c7

**Published:** 2026-08-07

**Authors:** Daniela P Valdés, Sarah N Zammataro, Andrii Melnyk, Ambar C Velazquez-Albino, Sk Rajab Ali, Carlos M Rinaldi-Ramos

**Affiliations:** 1 Department of Chemical Engineering, University of Florida, Gainesville, FL 32611, United States of America; 2 J. Crayton Pruitt Department of Biomedical Engineering, University of Florida, Gainesville, FL 32611, United States of America

**Keywords:** magnetic particle imaging, 3D printing, magnetic nanoparticles, phantom, quantification, segmentation

## Abstract

*Objective.* Many applications of magnetic nanoparticles (MNPs) require accumulation at target organs that can be challenging to achieve and to characterize. Magnetic particle imaging (MPI) enables sensitive and quantitative detection of MNPs, however the proximity of target signals to high accumulation organs hinders accurate quantification due to signal spillover effects. Specifically, we sought to systematically evaluate MPI quantification strategies under conditions mimicking pre-clinical animal studies and account for spillover. *Approach.* We developed an anatomically accurate 3D-printed mouse phantom with a fillable liver cavity, brain and lung ports and a hind flank cavity. By emulating high liver MNP uptake alongside low MNP concentrations in target locations at varying distances, we compared quantification from threshold-based and constant-volume segmentations, as well as a subtraction approach, both for 2D and 3D MPI scans. *Main results.* Thresholding was reliable for isolated high-signal regions but overestimated signal near the liver or at low signal-to-noise ratios. Constant-volume segmentation improved signal separation, and subtraction strategies mitigated spillover overestimation, enhancing the limit of quantification. With the subtraction approach we were able to quantify as low a signal as 0.05 and 0.25% of the total dose in the phantom in the brain/lung and hind flank of the mouse phantom with 3D MPI, respectively. *Significance.* These results underscore the importance of accounting for superimposed signals in quantitative MPI and highlight anatomically correct phantoms as essential tools for refining biodistribution assessment methods in nanomedicine using MPI.

**Abbreviations**
2D: two-dimensional
3D: three-dimensional
CAD: computer aided design
CT: computed tomography
CV: Constant Volume
CVS: Constant Volume with Subtraction
FOV: field of view
FFR: field-free region
ID: injected dose
MNP: magnetic nanoparticle
MPI: magnetic particle imaging
MRI: magnetic resonance imaging
NAMs: new approach methodologies
PET: positron emission tomography
ROI: region of interest
SNR: signal-to-noise ratio
SPECT: single-photon emission computed tomography
nDPEG: nitroDOPA-polyethylene glycol
THF: tetrahydrofuran

## 1. Introduction

Nanoparticle drug delivery holds significant promise for cancer and other diseases by enabling temporally and spatially controlled delivery of therapeutics with reduced systemic toxicity (Luo 2023). However, efficient transport and accumulation at the target site remain the primary obstacles limiting its clinical impact (Coelho *et al* 2010). Most systemically administered nanoparticles are sequestered by the mononuclear phagocyte system and eventually accumulate in the liver and spleen (Ngo *et al* 2022), while only a small portion reach target regions, such as small tumors. In this context, meta-analyses have shown that a median of only $\sim$0.7% of the injected dose (ID) reaches solid tumors, largely independent of nanoparticle size, shape, composition, or surface chemistry (Wilhelm *et al* 2016, Cheng *et al* 2020, Chen *et al* 2023). Importantly, these same meta-analyses reveal a gap in systematic, quantitative evaluation of nanoparticle biodistribution dynamics: only a small fraction of studies report quantitative biodistribution data at three or more time points (Chen *et al* 2023). This highlights the need for improved tools and methodologies to rigorously assess and overcome barriers to effective nanomedicine delivery.

Non-invasive quantitative imaging techniques are powerful tools for evaluating nanoparticle biodistribution dynamics. Both magnetic resonance imaging (MRI) and nuclear imaging, such as positron emission tomography (PET) and single-photon emission computed tomography (SPECT), allow for longitudinal studies *in vivo* but have significant drawbacks in studying nanoparticle biodistribution quantitatively. MRI contrast is indirect, arising from magnetic nanoparticle (MNP) or gadolinium-based agents that modulate water proton relaxation times, producing T2 or T1 contrast, respectively (Estelrich *et al* 2015). The technique might not be entirely quantitative due to this modulation also depending on tissue contrast (Lind *et al* 2017) and requires the use of specific acquisition sequences or analysis techniques (Hoopes *et al* 2012, Lind *et al* 2017). Although PET/SPECT offer high sensitivity and quantitative capability, they rely on short-lived radiolabels produced in cyclotron facilities (Skotland *et al* 2022) and assume stable label-nanoparticle coupling. Any radiolabel dissociation leads to off-target uptake, such that the measured biodistribution reflects the radioisotope rather than the true *in vivo* fate of the MNPs (Chakravarty *et al* 2017). Fluorescence imaging is a simple and widely available alternative but is greatly affected by tissue-dependent attenuation (Liu *et al* 2012) and signal distortion due to quenching, dequenching and saturation of the fluorophores used that hinder quantification (Meng *et al* 2018).

Magnetic particle imaging (MPI) is an emerging imaging modality that leverages the unique magnetic response of engineered MNPs to generate the image. It relies on a strong selection gradient magnetic field to generate regions of MNP saturation, and a small field-free region (FFR) where the MNP can respond to a weaker uniform alternating magnetic field. This response generates a signal in the MPI receive coils, while MNPs outside the FFR do not (Knopp and Buzug 2012). To generate an image, the FFR is scanned across the field of view (FOV), and the result is a map of signal intensity, which is directly proportional to the mass of MNP in a particular location. Most importantly, MPI does not display tissue attenuation and is quantitative in nature, so it can be used to produce MNP biodistribution maps non-invasively *in vivo* and throughout timepoints. Its attributes of negligible tissue attenuation and background signal, linear quantitative behavior, lack of ionizing radiation, and high sensitivity make MPI a promising modality for the evaluation of nanoparticle biodistribution. However, MPI resolution is currently in the order of 1–2 mm, similar to PET/SPECT, making it challenging to discern nearby signals within a FOV (Fink *et al* 2023, Fernando *et al* 2024). This poses challenges when applying MPI for *in vivo* studies, especially in small animals like mice.

Recent articles on MPI signal identification, separation and quantification have pointed out a phenomenon known as ‘shine-through’ or ‘spillover’ that must be accounted for to improve accuracy and reliability of quantitative MPI (Sehl *et al* 2024, Shakeri-Zadeh *et al* 2025). This phenomenon, which is also prevalent in MRI (Gatidis *et al* 2015, Perri *et al* 2015, Duran *et al* 2014) and nuclear imaging (Abdul-Fatah *et al* 2009, Kolb *et al* 2015, den Toom *et al* 2020), refers to the leakage of signal from adjacent areas within a region of interest (ROI), which significantly affects *in vivo* quantification of target sites adjacent to vasculature in early timepoints or adjacent to large accumulation organs like the liver for longer timepoints after systemic administration of the tracer (Shakeri-Zadeh *et al* 2025). Shakeri-Zadeh and coauthors analyzed the spillover effect on MPI images in controlled phantoms, where they calculated the total signal for fiducials at different distances from more-concentrated targets. They further evaluated spillover *in vivo* in a live mouse subjected to three consecutive systemic tracer administrations through a tail catheter at 40 min intervals, leading to saturated liver/spleen macrophage uptake. They prepared two fiducials with different amounts of tracer and placed them in different positions with respect to the accumulated signal, yielding an unintended signal increment in the fiducials due to spillover from the liver/spleen. While these authors studied signal increment due to spillover in terms of signal and acknowledge that it would result in overquantification, they do not transform signal values into mass, nor do they suggest a strategy to counteract the spillover. Rather, they affirm that appropriate calibration and optimization can preserve MPI’s quantitative accuracy, and that the MPI community needs to develop methods to account for and further reduce spillover effects.

Anatomically correct phantoms serve as valuable tools in preclinical research, allowing for the simulation of imaging conditions without the need for live animal experimentation (Wegner *et al* 2023), advancing the 3R principles promoting the ethical use of animals in scientific research (MacArthur Clark 2017). Furthermore, these phantoms can be produced via additive manufacturing, providing accurate anatomical structures while reducing the cost of tooling (Wegner *et al* 2023). Our group previously reported the design and use of a 3D-printed mouse phantom to investigate the impact of magnetic tracer accumulation in the liver on MPI sensitivity in brain and breast tumor models (Sarna *et al* 2022). That 3D-printed phantom was developed using computer aided design (CAD) techniques to extract the mouse anatomy from the Digimouse 3D whole body mouse atlas surface tessellation data (Dogdas *et al* 2007). In this article (Sarna *et al* 2022) the authors reported quantification of signal in the brain and breast tumor insert in the presence of a liver cavity filled with 10 and 200 $\mu$gFe, respectively. They discussed difficulties that arise due to the presence of the liver signal, such as identifying and thresholding signals in the ROI, as well as their impact on the MPI limit of detection. However, their study did not systematically evaluate the effect of liver signal when varying the distance with respect to the target site nor did they propose strategies to improve quantification accuracy.

In this work we report a new design for the 3D-printed mouse phantom that consists of a fillable liver cavity, ports to place capillaries in the brain and lung regions, and a hind-flank cavity. We acquired images of the phantom emulating situations where high liver uptake coexists with low MNP accumulation in target regions. We considered scenarios representative of different tumors: (i) small, internal and far from the liver; (ii) superficial, spatially extended and far from the liver; and (iii) localized near the liver, where spillover is expected to be most severe. We tested quantification using different segmentation approaches, such as threshold-based and constant volume spherical segments and we developed a subtraction strategy to mitigate liver-induced spillover.

## 2. Materials and methods

### 2.1. Materials

For the synthesis of the MNPs, an iron(III) oleate precursor was prepared in lab from iron(III) acetylacetonate ($>$98% pure) purchased from TCI American and oleic acid (90% technical grade) purchased from Sigma-Aldrich. Other materials involved in the synthesis and washing procedures were oleyl alcohol (80%–85% technical grade), hexane ($>$98.5%, certified ACS), toluene ($>$99.5%, certified ACS), ethanol (200 proof), and tetrahydrofuran (THF, 99.8% for HPLC) purchased from Thermo Fisher Scientific.

For the coating of the MNPs, the coating polymer molecule was synthesized using methoxy polyethylene glycol with MW of 5kDa purchased from JenKem USA, 3,4-Dihydroxy-L-phenylalanine ($>$98%), N-Hydroxysuccinimide, and chloroform were purchased from Sigma-Aldrich. Sodium nitrite was purchased from Fisher Scientific and N,N’-Dicyclohexylcarbodiimide was purchased from Thermo Fisher Scientific.

For the iron quantification in the MNP stock, nitric acid (67%–70%, for ultra trace elemental analysis) and hydroxylamine hydrochloride ($>$99%) were purchased from Thermo Fisher Scientific and 1,10-phenanthroline monohydrate ($>$99.5%, ACS certified) was purchased from Sigma-Aldrich.

For 3D printing, Clear V4 Resin was purchased from Formlabs Inc. and isopropanol ($>$99.9% for HPLC) from Thermo Fisher Scientific.

### 2.2. MNP synthesis

The MNP cores were synthesized through an esterification process described in Velazquez-Albino *et al* (2026). Particularly, 13 ml of oleyl alcohol were added to a 100 ml three-neck flask, placed in a heating mantle, and mixed using a magnetic stir bar and plate. The reactor’s left neck was connected to a Schlenk line, and the middle neck had a rubber septum holding a thermocouple used to monitor the temperature throughout. A glass stopper was added to the right neck of the reactor before starting a vacuum treatment for 1 h while heating to 140 $^\circ$C. After the vacuum treatment, the reactor was purged with Argon. Then, the reactor was wrapped in insulation before heating to the reaction temperature of 320 $^\circ$C. After reaching the aforementioned temperature, the iron oleate was dripped from the middle neck of the reactor at 0.16 ml min$^ {-1}$ using a syringe pump. MNP crude was washed prior to polymer coating using a solvent/anti-solvent wash procedure with magnetic separation in a Halbach array. Specifically, 1 ml of the crude from the reaction mixture was added to a 15 ml centrifuge tube with 0.3 ml of hexane and 1 ml of ethanol. The solution was vortexed and placed in a Halbach array for 10 min. Following, the supernatant was decanted and the MNPs were resuspended into 1 ml of hexane along with 1 $\mu$l of oleic acid. The solution was sonicated for 1 min before ethanol was added and the mixture was placed back in the Halbach array. This was repeated one time further and the final solution was resuspended in THF.

MNPs were imaged using a FEI Talos F200i S/TEM at 200 kV. Washed MNP solution was dropped onto a 200-mesh carbon-coated copper grid. Images were analyzed using MATLAB code to determine the diameter of over 1000 particles to generate a diameter histogram. A representative transmission electron microscopy image of the synthesized particles can be seen in figure S1(A) in the supplementary material, along with the histogram in figure S1(B). Their average diameter is 19 nm.

### 2.3. MNP coating

Polymer coating of the MNPs was carried out using a ligand exchange method. Therein, oleic-acid stabilized iron oxide nanoparticles were coated with an in-lab synthesized polyethylene glycol-based polymer possessing a 6-nitro-L-3,4-dihydroxyphenylalanine as an anchor group (nDPEG). The nDPEG ligand was synthesized as reported in the literature with slight modification (Napolitano *et al* 1992, Amstad *et al* 2009). Due to its high affinity towards the iron oxide MNPs surface, the nDPEG ligand conjugated to the particles, enhancing their colloidal stability. For this purpose, 4 mg of oleic-acid stabilized MNPs in 0.4 ml toluene was added to a solution of 22 mg of nDPEG in 4.6 ml toluene prepared by heating at 100 $^\circ$C in a 15 ml Pyrex tube. After probe sonication (750 W, 35% amplitude, 1 min), the particle solution placed on a heating block at 100 $^\circ$C for 24 h. Next, after cooling down the solution, 15 ml of diethyl ether were added, and the nDPEG-coated MNPs were magnetically separated using a Halbach array. After, the MNPs are redispersed in 4 ml of THF with probe sonication (750 W, 35% amplitude, 1 min) and purified by repeatedly washing with diethyl ether and separating using the Halbach array. Finally, the nDPEG-coated MNPs were dispersed in deionized (DI) water and further purified by passing the solution through a Miltenyi magnetic column.

### 2.4. Iron quantification

The iron concentration for the nDPEG-coated MNP stock was determined through the 1,10- phenanthroline assay, following a similar procedure to the one reported in the standard (ASTM Committee E15 on Industrial and Speciality 2004). In particular, 10 $\mu$l of MNP solution in DI water were digested in 70% nitric acid overnight in a heating block at 100 $^\circ$C, in triplicate. Following digestion, 10 $\mu$l of each replicate was placed in a quartz plate and liquid was evaporated in a heating block at 115 $^\circ$C. The quartz plate was placed in the Opentrons OT-2 Liquid Handler to complete the assay, where 46 $\mu$l of DI water was added to each replicate well, followed by 30 $\mu$l of hydroxylamine solution. An hour after hydroxylamine addition, the liquid handler added 49 $\mu$l of sodium acetate solution and 75 $\mu$l of 1,10-phenanthroline, for a total of 200 $\mu$l of liquid in each well. Simultaneously, reference samples with known iron masses were prepared and underwent the same procedure in the robot. The plate is removed from the robot and placed in the Spectramax-M5 Plate Reader, and the end-point absorbance is measured at 508 nm. Concentrations for each nDPEG-coated MNP aliquot were determined by relating the absorbance to that of the reference samples.

### 2.5. MPI performance

To determine the coated MNP performance in MPI, we used a MOMENTUM MPI Scanner (Magnetic Insight, Alameda, CA, USA) in the Relax mode and fitted it to a Langevin derivative model to determine the signal intensity and full-width-at-half-maximum (FWHM), relating to the sensitivity and resolution of the MNPs, respectively. A 10 $\mu$l aliquot of MNP solution was loaded in a microcentrifuge tube and scanned in this mode. The results can be seen in figure S1(C) of the supplementary material. The signal intensity obtained from the fits was 141 mgFe$^ {-1}$ and the FWHM 8.5 mT.

### 2.6. 3D-printed phantom design

This study utilized existing CAD files (Sarna *et al* 2022) of the Digimouse 3D whole body mouse atlas reported by Dogdas *et al* (2007) to facilitate the creation of a custom mouse phantom in OnShape (OnShape, Inc.). The initial design leveraged CAD techniques to generate 3D-printable models of the mouse anatomy using surface tessellation data. The original body and organ CAD geometries were reused and independently modified in this work to create a new phantom design. The external mouse body was segmented into head, torso, and hindquarter components using two transverse planes and the Split tool. Alignment pegs and corresponding holes were added using extruded circular sketches to enable repeatable assembly and positioning within the animal bed.

The liver cavity was created by subtracting the anatomical liver geometry from the torso segment. Fill ports and air vents were added using Extrude-Remove operations to allow the cavity to be filled with tracer solution while permitting air to escape during filling. In contrast, the brain and lung regions are represented by cylindrical capillary channels created using Extrude-Remove operations. These channels were positioned through the centers of the corresponding anatomical structures to provide anatomically accurate horizontal placement of capillary tube samples. A capillary filling guide was also designed to ensure consistent vertical positioning of the tracer sample.

Two hindquarter designs were developed. The first consisted solely of the hindquarter segment and was used for brain and lung imaging studies. The second incorporated a removable flank tumor insert. The tumor insert was designed as a spherical cavity enclosed within a solid body and included fill ports and air vents for tracer loading. To ensure repeatable positioning, a cross-shaped alignment feature was added to the insert using the Extrude tool, and a corresponding cavity with a small clearance tolerance was removed from the hindquarter segment using Extrude-Remove. All models described can be found in the data repository associated with this article or in the lab’s GitHub repository.

### 2.7. 3D print processing

All CAD files were exported as STLs from OnShape and formatted for printing using PreForm (Formlabs Inc.). Each model was 3D printed with a Form 3 stereolithography printer (Formlabs Inc.) using Clear V4 resin with a layer size of 0.100 mm for the bed and 0.050 mm for the models. During the 3D-print processing procedure, which consists of soaking prints in an isopropanol bath and curing the resin, all cavities were manually cleared of resin. Cavities with narrow openings, such as the liver and flank tumor cavities, pose difficulties in resin removal. This leads to inconsistencies in the true volumes of each model. To address this, the resulting models were characterized by filling the cavities with DI water and measuring the mass of the model before and after filling. The average difference in mass resulted in average loaded mass, which directly correlates to the average volume as the density of water is 1 g ml$^ {-1}$. These average volume measurements were later utilized in dilution calculations. We obtained an average flank volume of 65 $\mu$l with a standard deviation of 3 $\mu$l and an average liver cavity of 830.5 $\mu$l with a standard deviation of 0.4 $\mu$l.

The resin is diamagnetic, with a susceptibility close to that of water (Sangal *et al* 2023) and scans of 3D printed parts devoid of MPI tracer confirm that there is no additional signal relative to empty bore scans. Once excess resin was removed, the 3D prints were cured in a FormCure for 15 min at 60 $^\circ$C. Following the processing, each model was scanned in the MOMENTUM MPI Scanner (Magnetic Insight, Alameda, CA, USA) in 2D High Sensitivity (3.0 T m$^ {-1}$ gradient strength) to ensure no magnetic contamination on the prints.

### 2.8. Cavity and capillary filling

A single dilution at 0.12 mgFe ml$^ {-1}$ was prepared for filling the liver part, allowing for the filling of the 830.5 $\mu$l cavity with $\sim$100 $\mu$gFe. The dilution was placed into the model with a 1000 $\mu$l pipette and special attention was put into eliminating air bubbles through the air vent by gently tapping the model against the preparation surface. Once filled, a ball of putty (Chā-Seal, Kimble) was gently pressed on the fill port and air vent to seal the model and prevent leaks.

For the brain and lung samples, a serial dilution of a 0.98 mgF ml$^ {-1}$ stock was prepared with gradually decreasing iron masses ranging from 2.5 to 0.05 $\mu$gFe. Capillary tubes with internal diameters of 0.8 mm were filled with around 2.5 $\mu$l of each dilution using a 2.5 $\mu$l pipette. Each sample’s position was adjusted using the 3D-printed capillary filling guide displayed in figure S2 in the supplementary material to ensure the sample was contained inside the phantom once placed into the corresponding capillary hole. All the capillaries were sealed on both ends with Chā-Seal and the sample position verified again using the capillary filling guide in case movement was promoted by the sealing process.

The flank tumor samples were prepared through the serial dilution of a 0.038 mgFe ml$^ {-1}$ stock. The final samples had gradually decreasing iron masses ranging from 2.5 to 0.05 $\mu$gFe in 65 $\mu$l of DI water. The model was filled with a 100 $\mu$l pipette, tapped to remove air bubbles and sealed with Chā-Seal in fill port and air vent. The initial concentrations of each serial dilution were calculated so we could fill the highest test mass capillary or cavity without diluting.

Reference curve samples were created with varying masses with each volume sample having its own respective reference curve due to previous reports stating that quantification accuracy depends on matching reference and sample volumes (Zammataro *et al* 2025). The 2.5 $\mu$l sample (brain and lung) reference curve consisted of eight samples with iron masses 0, 0.05, 0.1, 0.25, 0.5, 1, 1.75 and 2.5 $\mu$gFe in 2.5 $\mu$l filled capillary tubes. The 65 $\mu$l flank tumor reference curve also consisted of eight samples with iron masses 0, 0.05, 0.1, 0.25, 0.5, 1, 1.75 and 2.5 $\mu$gFe in 65 $\mu$l flank cavities.

All ROI and reference samples were prepared the day of imaging to ensure the correct concentration.

### 2.9. Image acquisition

3D MPI scans were obtained on the MOMENTUM MPI Scanner (Magnetic Insight, Alameda, CA, USA) in standard mode (5.7 T m$^ {-1}$ gradient strength) for all sample configurations. The 3D scans were acquired utilizing 35 projections in the 6 ($x$), 6 ($y$) and 12 ($z$) cm full FOV, with a 0.3 cm offset in the $z$ direction, to encompass the entire phantom. After each 3D MPI scans, computed tomography (CT) scans were collected on an IVIS Spectrum CT (Perkin Elmer), maintaining phantom position consistent.

In particular, the phantom was imaged with gradually decreasing iron mass in each ROI (brain, flank and lung) and a liver cavity filled with 100 $\mu$gFe (what we call ‘subject scans’). The BR2 and LU2 holes were used to place the capillaries into for the brain and lung dataset, correspondingly. Also, a phantom with an empty ROI and just the liver cavity filled was imaged each day of the study (‘reference scan’). The reference samples and an image registration dataset were imaged in the same conditions. The registration dataset consisted of a 3D MPI and a CT scan of four fiducials with MNP solution in 1:1 DI water and Omnipaque CT contrast agent (GE HealthCare) taped into the bed (two in the $y$ and two in the $z$ direction). The time for MPI acquisition was $\sim$50 min for each 3D scan.

### 2.10. Image analysis

The analysis of all MPI scans was conducted using 3D Slicer (Slicer 5.8.1), an open-source software for medical image analysis (Kikinis *et al* 2013, BWH and 3D Slicer contributors), complemented by Python scripts. The MPI data acquired consisted of 35 projections obtained through an X-space direct reconstruction and the application of a manufacturer-implemented equalization filter (Lu *et al* 2013). Reconstructed volumes were obtained from the scanner’s implementation of filtered back-projection with default parameters and no additional filtering or post-processing was applied. The data was analyzed as 3D volumes (filtered back projection reconstruction from the 35 projections) or 2D volumes by taking just the center projection of the 3D scan (17th projection). Linear transformations were performed to the MPI and CT volumes to display the data in the correct coordinate system.

Slicer’s Landmark Registration Tool was used to obtain the registration matrices for the study. The process consisted in colocalizing the four fiducials in the CT scan from the registration dataset with their signal in the corresponding MPI images (both as 3D and 2D volumes).

A Python script to segment and quantify signals from the MPI phantom data was created. The script was run in Slicer’s Python console. An overview of the analysis pipeline done with the script can be seen in figure S3 in the supplementary material. The input is a comma-separated value table, detailing each MPI scan label (i.e. ROI and iron mass) and path, as well as the corresponding CT scan path for that configuration. The script prompts the user for the desired analysis mode (3D or 2D MPI volume) and registers the MPI volume with its CT scan by applying the corresponding registration matrix. It creates a mouse contour segment from the high-pass thresholding of the phantom-mouse body from the CT, as well as a liver segmentation from the MPI volume by searching for the maximum signal ($S_{\mathrm{max}}$) in the liver region and thresholding all the voxels with values from 0.5$S_{\mathrm{max}}$ to $S_{\mathrm{max}}$ (0.5Max Threshold). These two segments provide a spatial reference when displayed with the MPI scan.

To obtain segmentations of the ROIs being considered (i.e. brain, flank or lung), the user is prompted to enclose the ROI region with an interactive-boundary box and a search for the maximum value is performed inside the box. In this article we explore different segmentation approaches for the ROI signals: 0.5Max Threshold (Threshold), CV and constant volume with subtraction (CVS). Once segmented, the total signal $S$ for the ROI is calculated as the $S = N\langle s \rangle$, where $N$ is the number of voxels and $\langle s \rangle$ is the average signal in the segment.

To convert total signal to quantified iron mass of the ROI, the same segmentation approach is used to segment the reference samples with known iron mass and their nominal iron mass $m$ vs Total Signal $S$ is fitted with the linear regression $m [\mu\mathrm{g}] = a [\mu\mathrm{g}/\mathrm{arb.u.}]S[\mathrm{arb.u.}] + b [\mu\mathrm{g}]$, obtaining $a$ and $b$. This conversion equation is then used to transform any total signal to quantified iron mass. Note that we use reference samples with volumes similar to that of the ROI we want to quantify. For example, to obtain conversion equations for brain and lung, we obtain $a$ and $b$ for capillary reference samples while for flank we used 65 $\mu$l cavities as reference samples. Figure S4 in the supplementary material shows the nominal iron mass vs. total signal for the reference samples for all methods used to segment and table S1 has the fit parameters $a$ and $b$, as well as the $r^2$ value for the data.

## 3. Results

### 3.1. 3D-printed phantom design

**Anatomically-correct mouse phantoms with multiple fillable cavities enable the emulation of biologically-relevant scenarios**

The phantom design is based on previous models and its design is described in section 2.6. The anatomical structure of the mouse is divided into three parts: the head, the torso, and the hindquarters. This division was achieved using two transverse sections across the mouse. To improve ease of use, and allow interchangeability of parts, the phantom parts were designed to be connected using friction-fitting pegs, enabling the phantom to be handled as a unified object while retaining the modularity of its components.

**Figure 1.**
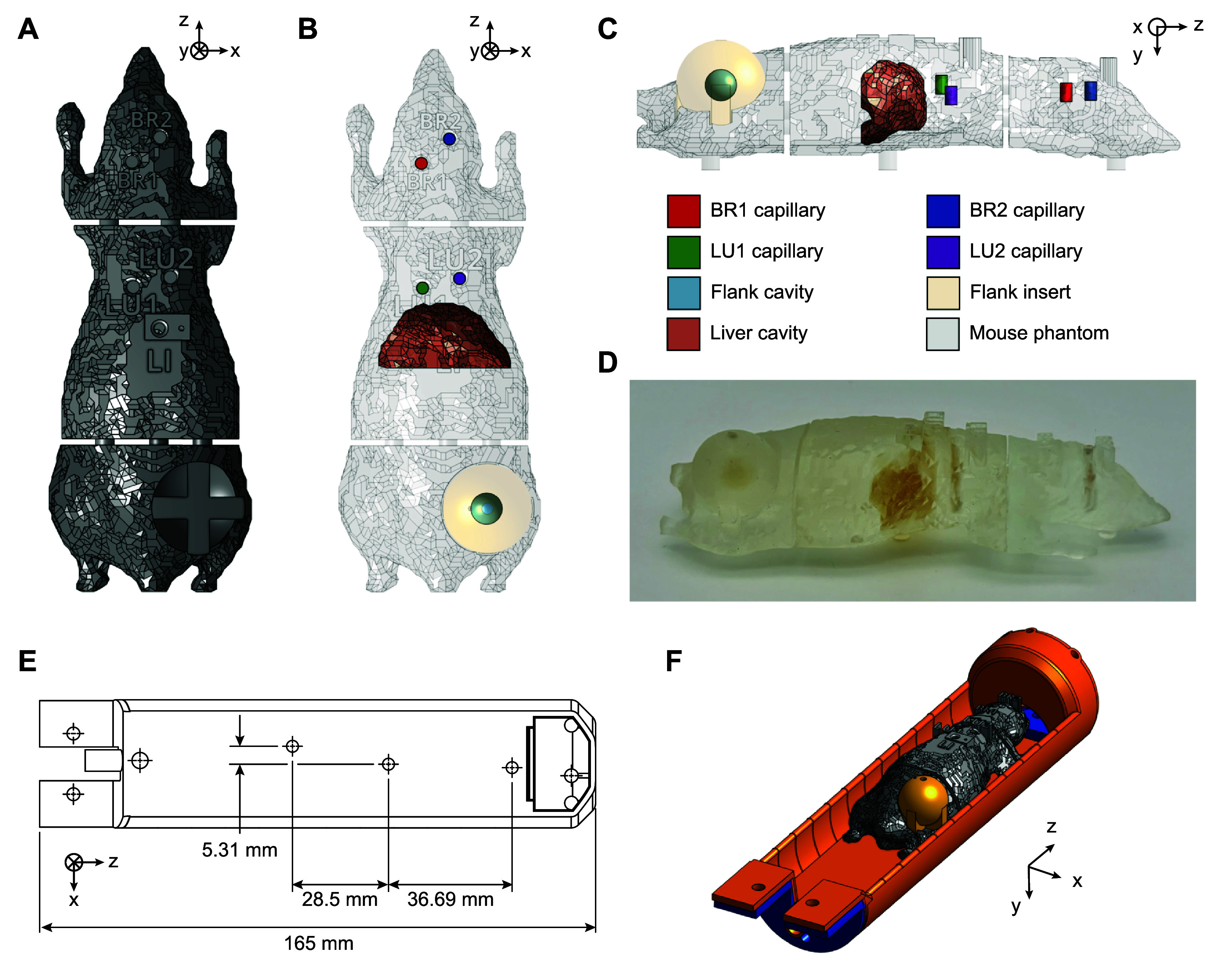
Additive manufacturing of mice phantoms enables the systematic study of biomedically relevant scenarios and consistent tracer positioning. (A) Solid and (B) transparent top views of the 3D-printed mouse phantom depicting regions of interest. (C) Side view of the transparent phantom illustrating locations for MNP tracer insertion. (D) Photograph of the 3D-printed phantom filled with a MNP solution containing 100 $\mu$gFe in the liver, 2.5 $\mu$gFe in the flank and two capillaries with 2.5 $\mu$gFe in the BR2 and LU2 positions. (E) Top view of a custom bed with holes that align with pegs on the bottom of the phantom, and (F) isometric view of the bed with the mouse phantom.

The head portion design includes two cylindrical cavities across the y axis each with a diameter of 1.9 mm. These cavities house tracer filled capillary tubes (0.8 mm inner $\times$ 1.6 mm outer diameter) to represent the two hemispheres of the brain. The left hemisphere is labeled BR1, and the right is labeled BR2 (see figures 1(A)–(C)).

The torso design incorporates two cylindrical cavities across the y axis, each with a diameter of 1.9 mm to accommodate a small capillary with a small volume of tracer that would represent a small ROI in either lung. The left lung is labeled LU1, and the right is labeled LU2. The torso also includes an anatomically correct cavity for the liver, labeled as LI. The cavity was created by subtracting the liver of the original CAD file from the torso part of the mouse phantom. To allow air to escape while the liver cavity is filled with tracer solution, a 0.8 mm diameter air vent was placed 20$^\circ$ relative to the liver fill port (see figures 1(A)–(C)).

There are two designs for the hindquarters portion of the phantom. The first design has no cutouts or cavities and was used when imaging the brain and lung ROIs. The second design includes a cutout fitted for a tumor insert to represent a subcutaneous melanoma tumor on the right hind flank region, used when imaging the flank tumor as the ROI. The flank tumor inserts were designed with different spherical cavity volumes to represent different stages of tumor growth. Like the liver cavity, the tumor cavity of the flank tumor insert is accompanied by a fill port and air vent to allow the air to escape while being filled. The flank tumor insert was also designed with alignment features for ease of assembly and consistent placement within the mouse phantom. While multiple flank tumors were designed, only the 65 $\mu$l flank tumor design was used for data collection (see figures 1(A)–(C)).

The 3D-printable animal bed reported by Liu and coauthors (Liu *et al* 2021) was adapted to allow for consistent placement of the 3D-printed mouse phantom (figure 1(D)). Holes with a 3.5 mm diameter were placed along the bed’s base. Aligned with the holes, pegs with diameters of 3.0 mm were extruded from the bottom of the mouse phantom. When assembled, the mouse phantom consistently sat within the bed in a headfirst prone orientation, typical of preclinical imaging in MPI studies as depicted in figure 1(F).

Along with the design used in this study, a modified design that contains anatomically-correct brain and lung fillable cavities can be found in the data repository for this article.

### 3.2. Imaging of the phantom

**Biomedically relevant scenarios often present quantification challenges, such as low target signals with respect to the background or target signals close to high accumulation sites**

We acquired 2D and 3D MPI images as described in section 2.9, emulating nanomedicine delivery relevant scenarios with the designed mouse phantom. For instance, the liver cavity was filled to contain the target amount of 100 $\mu$gFe, while smaller test iron masses were placed in different ROIs. The datasets were acquired for the iron masses of 0, 0.05, 0.1, 0.25, 0.5, 1, 1.75 and 2.5 $\mu$gFe in capillaries with 2.5 $\mu$l of solution for the brain (BR2 in figures 1(A)–(C)) and lung ROIs (LU2 in figures 1(A)–(C)) or in a cavity with 65 $\mu$l of solution for the flank ROI (Flank cavity in figure 1(C)). Throughout this article, we will correlate the phantom configuration being scanned with a % ID value, calculated as the nominal iron mass in the target region divided by the total nominal iron mass in the phantom (i.e. the sum of the target and liver contributions). The range for the masses was chosen to encompass from 0.05 to 2.44%ID in the target region, chosen due to its relevance to tumor delivery in cancer nanomedicine. The scenarios we are mimicking are those of terminal accumulation in the ROIs, as our motivation is MNP accumulation into tumors. Phantoms with MNP solution flow have been developed by other groups to evaluate applications that involve dynamics (Radon *et al* 2017, Reichl *et al* 2025).

**Figure 2.**
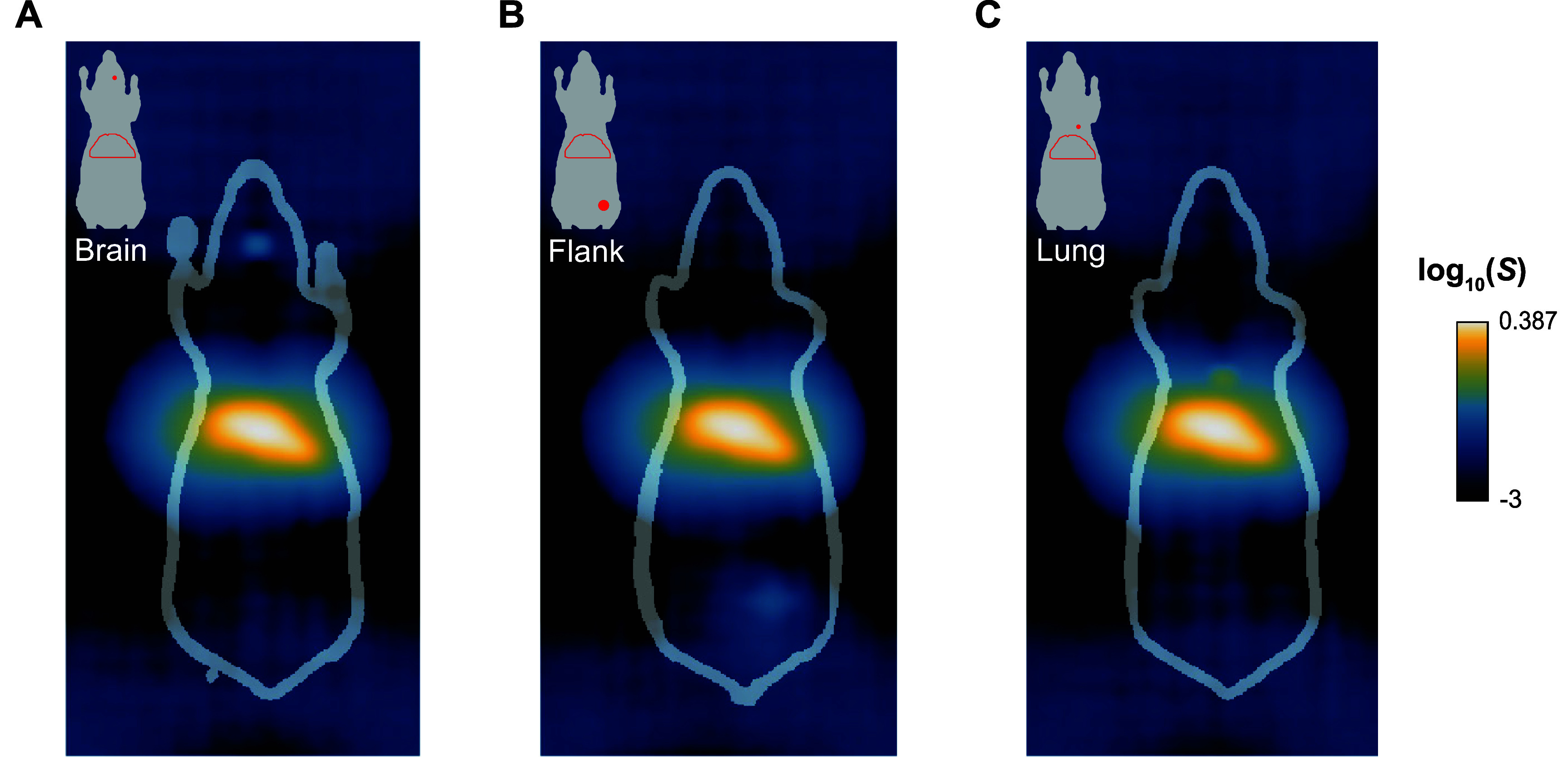
At fixed mass, signals in the brain and lung are localized but differ in terms of distance with respect to the liver, whereas signal in the hind flank is far from the liver but more spread-out. Logarithmically transformed MPI 2D volumes corresponding to (A) brain, (B) flank and (C) lung ROIs with 1.0 $\mu$gFe for a mouse phantom with 100 $\mu$gFe in the liver cavity. *Insets:* Model of the mouse phantom imaged. *Note:* Logarithmically transformed MPI signals are shown for illustrative purposes due to the large dynamic range of the MPI signal in these images.

The brain dataset has a very localized signal far away from the liver, emulating a small intracranial tumor or small region with internal bleeding, and serves as a case study where we expect the impact from the liver spillover to be minimal. The flank dataset mimics a superficial and relatively large tumor with signal more spread-out than in the brain case, which can pose challenges due to poor signal-to-noise (SNR) ratio. Finally, the lung dataset represents a small lung tumor and has again a very localized signal but close to the liver, which is severely affected by the spillover effect (Shakeri-Zadeh *et al* 2025). To illustrate these characteristics, a comparison of the three datasets in 2D, at a fixed mass of 1.0 $\mu$gFe (1% ID) and fixed color scale is presented in figure 2. For illustration purposes, these images use an MPI signal that has been transformed by taking the standard logarithm (logarithm with base 10) of each voxel to improve visualization in a wide signal dynamic range. Null values were saturated to black, which is also the color corresponding to the minimum of the scale. Note that all image segmentation and quantification operations presented throughout the article were carried out using the untransformed MPI signal.

Although both the brain (figure 2(A)) and lung (figure 2(C)) datasets correspond to a 0.8 mm inner diameter capillary filled with 1.0 $\mu$gFe, the signal values for the lung ROI are higher than those in the brain one, which could indicate that the signal decaying from the liver might be adding to that of the lung. The flank dataset corresponds to a cavity that has a larger diameter than the capillary (5 mm), which makes the signal more spread-out. Consequently, the signal values are lower and are close to the background. In fact, the peak SNR, calculated as the maximum value in the ROI divided by the standard deviation of an empty scan, is 44 for the brain signal in figure 2(A), 28 for the flank signal in figure 2(B) and 98 for the lung signal in figure 2(C). This confirms that, at a constant mass, the flank dataset signals are closer to the background and that the lung signal are unintentionally enhanced when compared to the brain’s.

### 3.3. Quantification from thresholded segments

**Thresholded signals produce accurate quantifications for highly-localized signals but fail when SNR ratio is poor or spillover is present**

**Figure 3.**
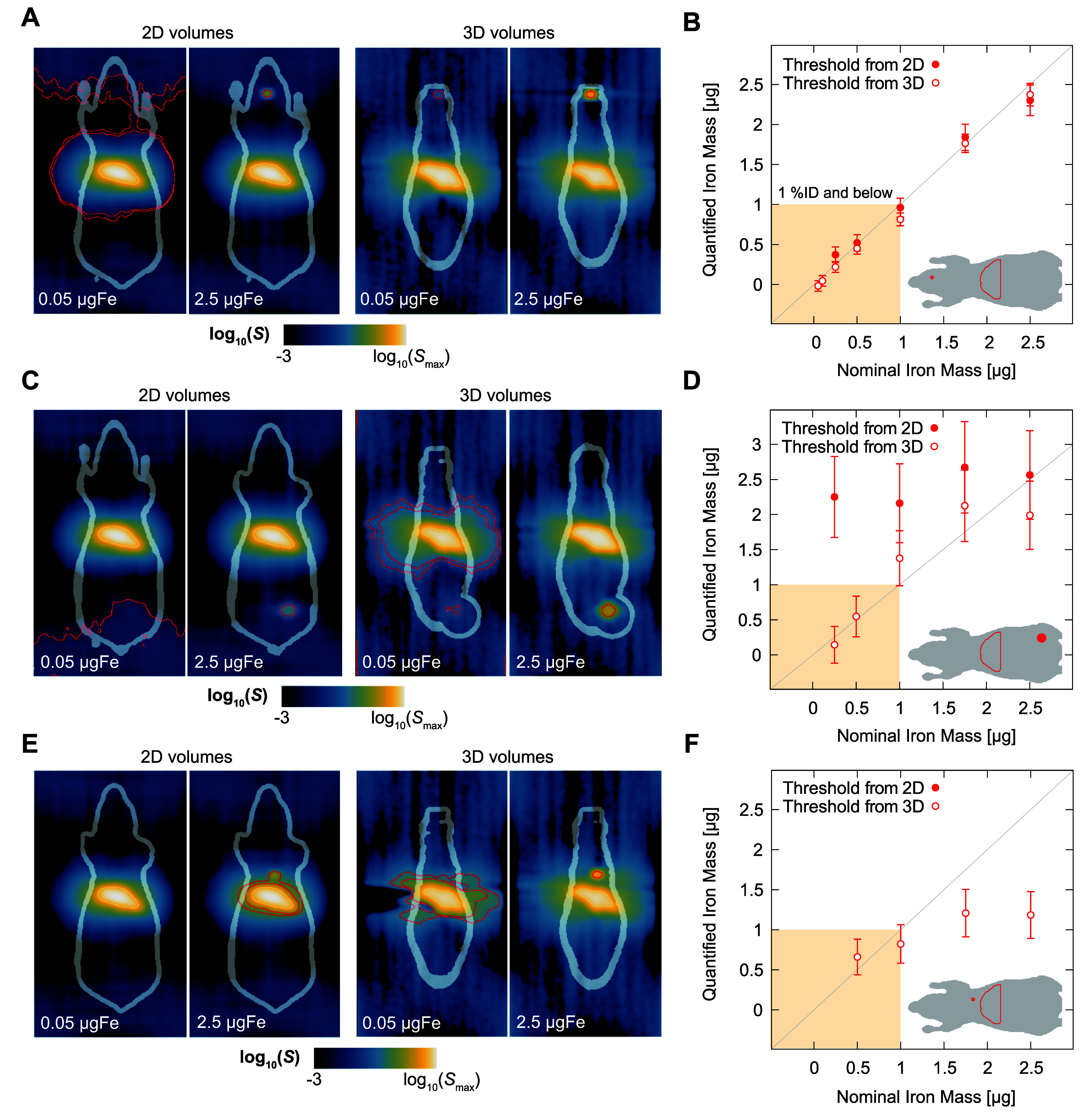
Thresholded segments allow the quantification of small, localized signals in the brain of a mouse below the 1%ID, but are challenging for more spread-out signals like in the flank or signals close to high off-target accumulation like lung-liver. (A) Slices of MPI volumes for a mouse phantom with 0.05 and 2.5 $\mu$gFe in the brain region coming from 2D and 3D volumes. (B) Quantified iron mass as a function of the nominal iron mass in the brain region for threshold segmentation. (C) Slices of MPI volumes for a mouse phantom with 0.05 and 2.5 $\mu$gFe in the flank region coming from 2D and 3D volumes. (D) Quantified iron mass as a function of the nominal iron mass in the flank region for threshold segmentation. (E) Slices of MPI volumes for a mouse phantom with 0.05 and 2.5 $\mu$gFe in the lung region coming from 2D and 3D volumes. (F) Quantified iron mass as a function of the nominal iron mass in the lung region for Threshold segmentation. The yellow shaded region highlights doses that represent 1%ID and lower values. *Note:* Logarithmically transformed MPI signals are shown for illustrative purposes due to the large dynamic range of the MPI signal in these images.

To quantify the signal in the ROIs, we need segmentations that provide a boundary in which quantification can be performed. The thresholding of signals is a simple segmentation method that has been used extensively in medical imaging (Xu *et al* 2024). When talking about ‘Threshold’ throughout the article we are referring to the selection of voxels with values lying in the range [0.5$S_{\mathrm{max}}$, $S_{\mathrm{max}}$], which is the 0.5Max criterion, with $S_{\mathrm{max}}$ being the maximum signal within the ROI. This approach is objective when there is clear signal separation between the target region and other signal sources but becomes subjective and challenging to apply when this is not the case (Hayat *et al* 2020, Sehl *et al* 2022). The MPI signals in the brain dataset were thresholded with this criterion, both from 2D and 3D volumes. The images in figure 3, as all the MPI scans shown in this manuscript, are logarithmically transformed for visualization purposes. The original images can be found in figure S5 in the supplementary material. The thresholds obtained for MPI volumes with an iron mass of 0.05 and 2.5 $\mu$gFe are shown with red outline in figure 3(A).

For 2D imaging, although the maximum corresponding to all the test iron masses in the brain could be localized, the threshold for the two smallest masses (0.05 and 0.1 $\mu$gFe) contains spurious signal. This is illustrated in figure 3(A) for a tracer mass of 0.05 $\mu$gFe in the brain, where the threshold segment includes the brain signal region, a ring around the liver, and parts of the background, illustrating the difficulty with threshold based segmentation for weak signal sources in the presence of strong signal sources. In contrast, for 3D imaging, the threshold segments contain just the brain signal source for all masses evaluated (figure 3(A)). While our analysis uses an interactive box to select the ROI in question and search for the maximum $S_{\mathrm{max}}$ inside of it, we allow the threshold to extend beyond the ROI to prevent arbitrary cutoffs from user interaction (see figure S6(A) in the supplementary material). Although there is signal around the liver that has values ranging from 0.5$S_{\mathrm{max}}$ to $S_{\mathrm{max}}$ in the 3D brain dataset, that part can be non-arbitrarily left out from the segmentation since, contrarily to the 2D case, it is not connected with the brain signal (see figure S6(B) in the supplementary material). This difference arises due to the SNR in 3D volumes being significantly higher than that from 2D images due to brain signal coming from different projections adding up during the reconstruction process.

Figure 3(b) shows the quantified iron mass as a function of the nominal iron mass placed in the capillaries in the brain region. These masses were quantified by considering the total signal inside the thresholded segments. Thresholds that extend artificially beyond the ROI in question (i.e. as the 0.05 $\mu$gFe shown in figure 3(A), 2D volumes) were excluded from the quantification as they will produce overestimation errors. For 2D, except for the 0.05 and 0.1 $\mu$gFe cases that could not be successfully thresholded, the quantification is close to the expected values except for 0.25 $\mu$gFe. While the 0.25 $\mu$gFe test mass was quantified as 0.37 $\mu$gFe, when considering the error the values extend from 0.27 to 0.47 $\mu$gFe, making the lower limit close to the ground truth value (see table S2 in the supplementary material). For 3D, while we could threshold the 0.05 and 0.1 $\mu$gFe cases, the values quantified are not distinguishable from 0 (see table S2 in the supplementary material). In conclusion, a localized signal in the brain of a mouse can be quantified from 0.5Max thresholds, from 0.5 $\mu$gFe (0.5%ID) and above for 2D and from 0.25 $\mu$gFe (0.25%ID) and above for 3D.

Analogously to the analysis done for the brain dataset, MPI signals in the flank and lung regions were thresholded with the 0.5$S_{\mathrm{max}}$ criterion and quantified as described in section 2.10, both as 2D and 3D volumes. The threshold segments obtained for MPI volumes with an iron mass of 0.05 and 2.5 $\mu$gFe are shown in figure 3(C) for the flank and in figure 3(E) for the lung dataset. The quantified vs nominal iron mass plots for the flank and lung thresholds are displayed in figures 3(D) and (F), respectively.

As in the brain dataset, all maxima could be localized for the flank but the thresholds for the smallest masses (0.05 and 0.1 $\mu$gFe) contain spurious signal from the background and around the liver. This time, the spurious signal in the thresholds happens even for 3D volumes (0.05 $\mu$gFe in figure 3(C), 3D volumes). Furthermore, quantification differs significantly when performed with thresholds from 2D or 3D volumes. For 2D, although the thresholds for test mass ranging from 0.25 to 1.75 $\mu$gFe are correctly localized in the flank area, the quantification yields overestimated values as evident in figure 3(D) and the values on table S3 in the supplementary material. The overestimated values suggest a systematic error affecting the mass quantification. As the signal values are lower and more spread out in the flank than in the brain dataset (see figure 2), signals being thresholded are close to noise levels. We hypothesize that part of the background noise might be contributing to the signal significantly or/and being included in the segments, yielding more signal than the one corresponding to just the MNPs filling the flank cavity. For 3D, test masses ranging from 0.25 to 2.5 $\mu$gFe were successfully quantified, although with high estimation uncertainty, making the 0.25 $\mu$gFe case indistinguishable from 0 $\mu$gFe. As the 3D volumes are reconstructed from thirty-five 2D projections acquired independently, the signal in the flank coming from all of them is added, contributing to an improved SNR for 3D imaging. In fact, the peak SNR for the flank signal in the 3D volumes gives 15–20 times the peak SNR in the 2D volumes. For reference, the smallest peak SNR in the dataset corresponds to the 0.1 $\mu$gFe test mass in 2D, being slightly above 8.

The lung dataset presents a very challenging case. For 2D, the two smallest masses (0.05 and 0.1 $\mu$gFe) were not detected and thus could not be thresholded and quantified. The threshold of the rest of the test masses (0.25 to 2.5 $\mu$gFe) produced indistinguishable signal between lung and liver signal decaying as the segment includes both the ROI signal and a ring around the liver (2.5 $\mu$gFe in figure, 3(E), 2D volumes). This means that there was no quantified data from 2D, even if the maxima can be located. For 3D, the signal could be successfully thresholded for test masses larger than 0.5 $\mu$gFe but the two largest test masses were underestimated, as seen in figure 3(F). Overall, the threshold method does not allow the quantification of signal in a mouse’s lung.

### 3.4. Constant volume segmentations

**Constant-Volume Segmentations enable detection of smaller signals when compared to thresholding but do not account for spillover effects**

We have identified problems for thresholding of the flank and lung signals, but the maxima could be localized in most cases. Although the flank and lung dataset difficulties differ in nature, both could benefit from the delimitation of the signal within a fixed-size segmentation centered in the maximum in question. Fixed-size segmentations have been previously used in MPI studies and are objective as long as the local maxima or anatomical feature can be localized (Liu *et al* 2021, Sehl *et al* 2022). This type of approach would cut part of the background out of the small mass segments for the flank and the signal coming from the liver for the lung dataset. This method is a common approach to segmentation from PET images and has already been reported in MPI (Sehl *et al* 2022).

**Figure 4.**
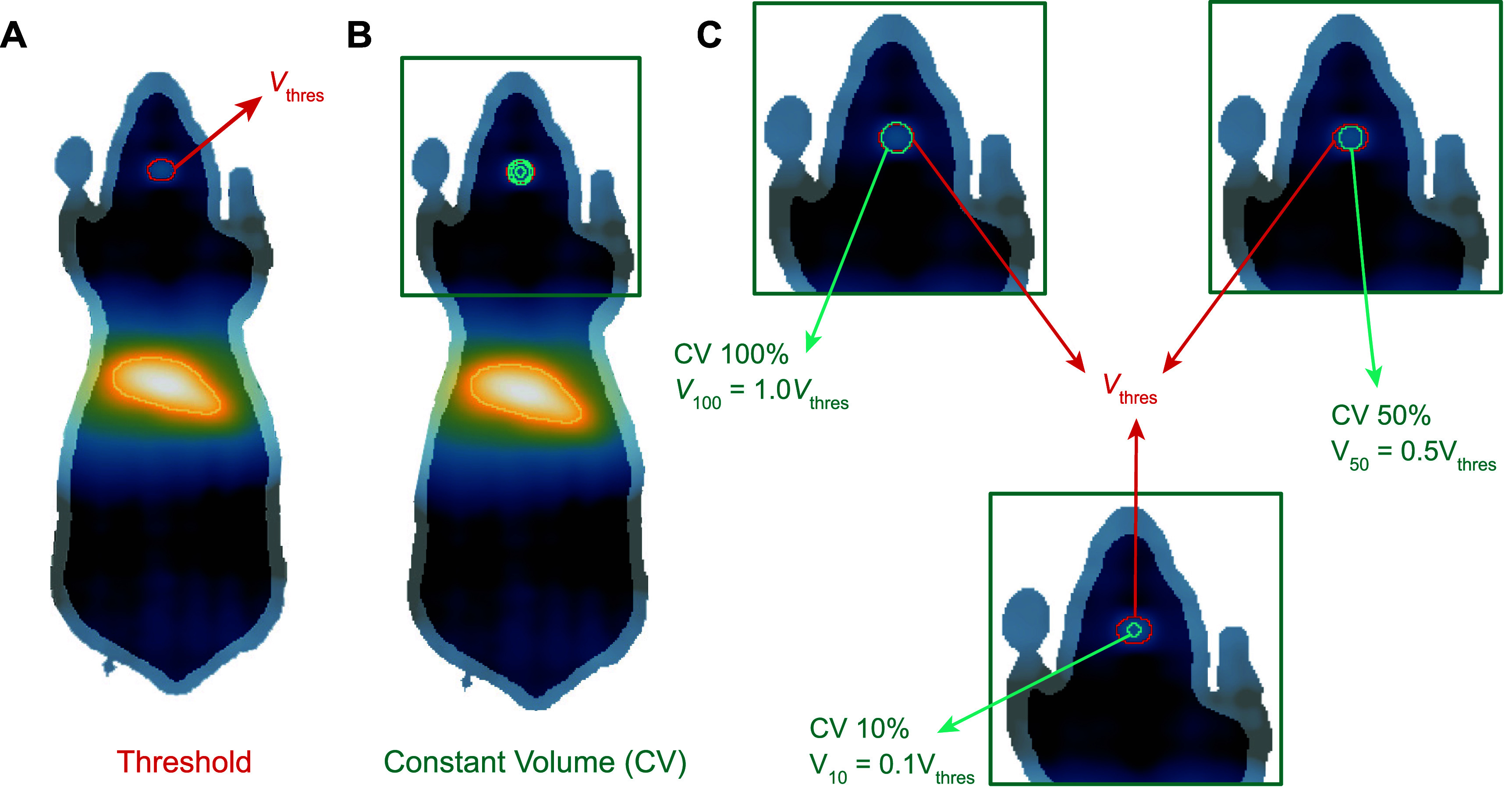
Anatomically-correct mice phantoms allow consistent positioning of signals and the testing of different segmentation methods such as thresholds and constant volume segments. (A) Thresholded segment corresponding to the 1%ID scan of the brain dataset (1 $\mu$gFe in the brain) and some of its corresponding spherical (B) Constant Volume segments (CV 100%, CV 50% and CV 10%). (C) Detailed comparison of thresholded segment with each of the different percentage CVs. *Note:* Logarithmically transformed MPI signals are shown for illustrative purposes due to the large dynamic range of the MPI signal in these images.

As we are particularly interested in accurately quantifying the scans corresponding to 1% ID and below, we defined the segmentation volume for our constant volume (CV) approach as the one obtained through the threshold of the 1% ID scan (i.e. the one for the 1 $\mu$gFe nominal mass) in each dataset, $V_\mathrm{thres}$ (see figure 4(A)), both in 2D and 3D. Then, we placed segments with spherical symmetry (i.e. spheres for 3D and circles for 2D, which represent cylindrical volumes when considering the slice thickness) centered in each ROI’s maximum with volumes $V_{10}$, $V_{20}$,$\ldots$,$V_{100}$ that are a fraction of the threshold volume, $V_i = \frac{i}{100}\ V_\mathrm{thres}$ (see figures 4(B) and (C)). After determining the volumes for the CV segmentations, we analyzed the scans in the corresponding dataset sequentially. The analysis consisted in selecting the ROI, searching for the maximum signal inside of it and inspecting the scan to determine if the maximum found corresponds to noise or if it is a detectable signal. The main giveaways of a non-detectable signal are: (i) a large local intensity gradient or lack of spatial continuity between the detected maximum and the surrounding background, (ii) a spatial misalignment between the position of the maximum found and the expected ‘anatomical’ location in the registered CT and (iii) a low SNR, making the peak intensity not significantly above background noise. If it is detectable, we place the segments with volumes $V_{10}$, $V_{20}$,$\ldots$,$V_{100}$ centered on the maximum. However, if it is not, we center the segments in the maximum position from the previous scan, since the placement of the signals in our phantom using the filling guide is consistent enough for the analysis. This allows analyzing scans where no signal peak is detected (i.e. for the smallest test masses).

Note that for the 1% ID scan we are using to determine the volumes for the CV segmentations, even if the CV 100% segment has a volume ${V_{100} = V}_\mathrm{thres}$, the symmetry is not the same. The threshold does not necessarily have spherical symmetry around the maximum, whereas we are forcing that symmetry onto the CV segments. This means that the quantified mass from the Threshold and CV 100% methods will be different because they do not include the same voxels. This is illustrated in figure 4, when comparing the shape of the thresholded segment (red contour) and the CV 100% segment (teal contour with largest diameter).

For the lung dataset, it is worth recalling that the thresholds from 2D could not be produced due to the signal being indistinguishable from the decay from the liver. Thus, we could not access $V_\mathrm{thres}$ from the volume of the 1% ID segment to calculate the CV needed for this analysis. As we are using capillaries of the same geometric proportions filled with the same test masses of MNP than in the brain dataset, just placed in another position in the phantom, we used the value for $V_\mathrm{thres}$ from that dataset. Although we had threshold data for the lung dataset in 3D, we also utilized the $V_\mathrm{thres}$ from the brain data set to eliminate discrepancy due to mismatches of the volumes used and to be able to compare 2D and 3D.

As the selection of the 1% ID scan to calculate the reference volume $V_\mathrm{thres}$ might seem arbitrary, we tested the different percentage CV segments as a way of accounting for the variability a different choice in reference volume might introduce. Specifically, the different percentage CV segments will contain different fractions of the total signal. In this section, we present the quantifications from CV 100% segmentations labeled as ‘CV’, while the results for the different percentages of $V_\mathrm{thres}$ can be seen in figure S7 of the supplementary material.

The brain dataset quantification, which was already close to the expected values, changes slightly by using the CV segmentation approach (see table S2 in the supplementary material). To be able to quantitatively assess the accuracy of our determinations, we use the relative error $\delta\left(m\right)$ with respect to the ground truth mass value, $m_\mathrm{GT}$, $\delta\left(m\right) = \left|\left(m-m_\mathrm{GT}\right)/m_\mathrm{GT}\right|$. For 2D, quantifications of the 2.5, 1.75, and 0.5 $\mu$gFe test masses stayed below 10% relative error. The 1 $\mu$gFe test mass increased its relative error from $-$4% to $-$12% but the 0.25 $\mu$gFe test mass reduced it from 48% to 20%. Moreover, the use of a fixed volume segmentation enabled the quantification of the two smallest test masses, although the relative error is high ($\unicode{x2A7E}$100%). For 3D, the 2.5, 1.75, and 0.5 $\mu$gFe test masses also stayed below 10% relative error. The 1 $\mu$gFe test mass increased its relative error from $-$19% to $-$27% but masses 0.25, 0.1 and 0.05 $\mu$gFe got quantified into the exact ground truth values (0% relative error). This represents an improvement if we consider that with the thresholded segments, 0.1 and 0.05 $\mu$gFe had more than 50% and more than 100% relative error, respectively. The relative errors are illustrated through the shading on table S2 in the supplementary material, for all methods and masses in the brain dataset.

**Figure 5.**
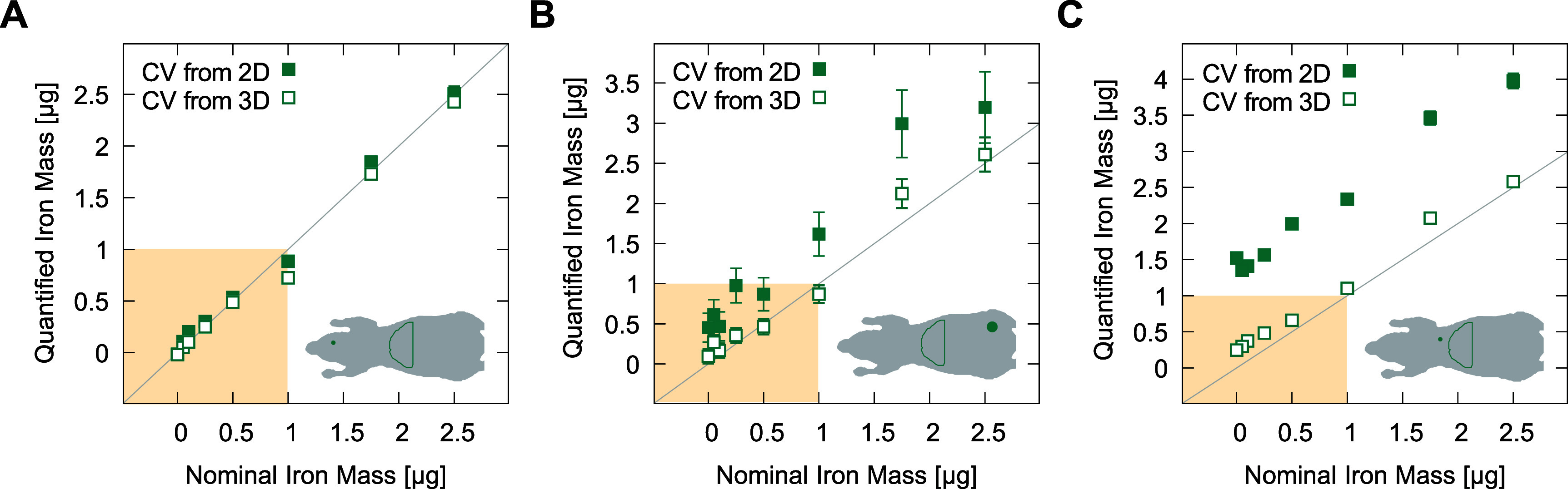
Use of constant volume segments at the maximum signal position enhances the limit of quantification for signals in the flank and lung of a mouse that also has strong liver accumulation. Quantified iron mass from the CV segmentation approach as a function of nominal iron mass for the (A) brain (B) flank and (C) lung datasets. The Threshold data is also presented to guide the discussion. The yellow shaded region highlights doses that represent 1%ID and lower values.

Now for the flank dataset, where we could not obtain thresholds for masses smaller than 0.25 $\mu$gFe, we could quantify the signal for all the volumes. For 2D, while the overall quantification was improved by the CV approach (see figure 5(B)), there still are overestimations showing up as an offset with respect to the expected-value trendline in our parity plots. For example, the 0.05 $\mu$gFe. mass is overestimated in 0.55 $\mu$gFe, which is approximately the overestimation for the 2.5 $\mu$gFe (see table S3 in the supplementary material). Considering that we now have the same controlled size for all segmentations, this could be a product of background being added to the signal. This effect was not present in the brain dataset, but it could be amplified here due to the signal being more spread out. For 3D, the quantification improves in the whole range, which would suggest that the origin of the overestimations for 2D is in fact poor SNR.

Figure 5(c) shows the quantified iron mass as a function of the nominal values obtained for the CV segmentation of the lung dataset. For 2D, while we can quantify smaller masses that we could not threshold before, the relative error in most of them is $\unicode{x2A7E}$ 100% for the CV approach (see shading in table S4 in the supplementary material). The plot shows the correct trend but it is drastically impacted by an offset, as we have previously detected for the flank dataset although with a smaller contribution. For 3D, we notice that there is an offset contributing to an overestimation of around 0.25 $\mu$gFe for all test masses except the largest (2.5 $\mu$gFe in table S4 of the supplementary material), which is quantified as (2.58 $\pm$ 0.05) $\mu$gFe. We consider this one to be an accurate quantification as the ground truth value is inside the range [2.53, 2.63] $\mu$gFe provided by the 0.05 $\mu$gFe estimation error.

When quantifying from the CV segments for fractions of the 1%ID-thresholded volume, the quantification varies negligibly for all datasets, which suggests that the change in signal distribution with nominal iron mass is not significant (see figures S4(A)–(F)). This means the analysis of the CV 100% in the main manuscript is applicable to the other segments taken for fractions of the 1%ID-thresholded volume too.

### 3.5. Constant volume with subtraction

**Subtraction strategies improve quantification from constant-volume segments and produce accurate quantifications even in high-spillover scenarios**

**Figure 6.**
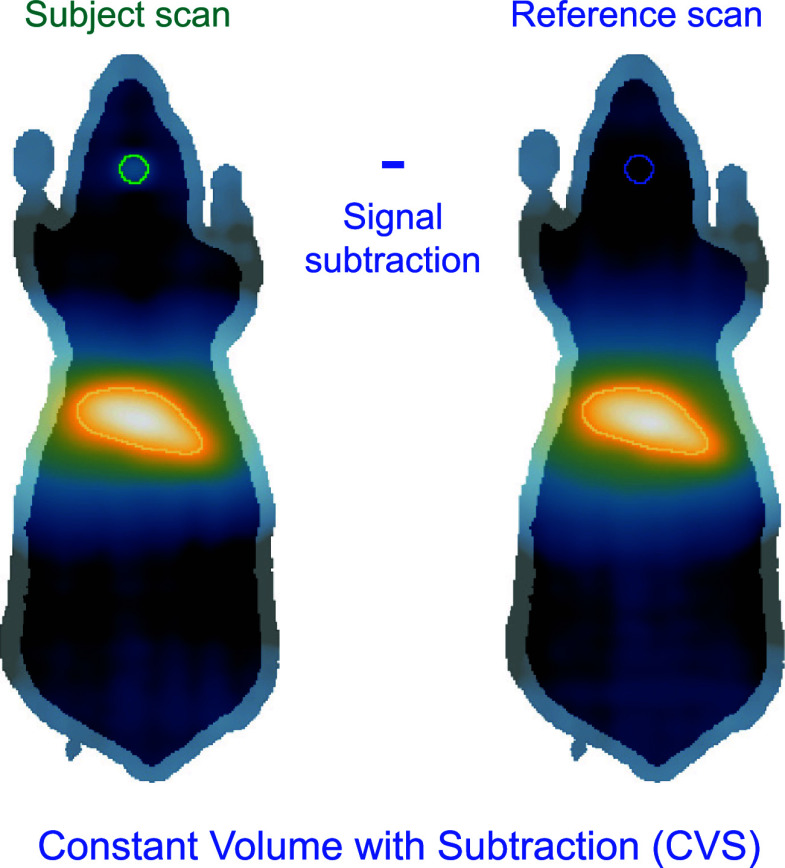
Anatomically correct mice phantoms allow the acquisition of reference scans for digital subtraction of spillover in target sites. Schematic representation of the constant volume with subtraction process, involving the CV 100% segment for the brain dataset in the 1.0 $\mu$gFe subject scan (filled capillary in the brain) and on the reference (just the liver cavity filled) scan.

Digital signal subtraction in MPI has been reported in previous papers (Yu *et al* 2017, Szwargulski *et al* 2020, Guan *et al* 2024). However, these subtractions were aimed at visualization of changes during longitudinal imaging and are tailored to filter for signal increments to visually detect MNP accumulation. These studies do not present quantification of the subtracted images, nor any other iron mass quantification. If background subtraction is to be applied within the ROIs we are analyzing prior to quantification, information on the signal that would be present at the same spatial location in the absence of ROI signal is required. The information we need for each test mass was obtained from the two phantom scans: (i) the subject scan, which we already analyzed, containing both the ROI signal (i.e. capillary in the brain/lung or filled flank cavity) and the filled liver cavity; and (ii) a reference scan, containing only the filled liver cavity. The CVS segmentation approach takes the spherical segments from the CV approach on the subject scan and uses those same segments in the reference scan (with just the liver cavity filled) to quantify the spillover signal in the ROI. Then, we subtract the spillover signal from the total signal obtained from the subject scan and quantify what would be the ROI signal if there were no spillover effect. This process is illustrated in figure 6.

In brief, as the reference scan contains no signal in the ROI but almost the same signal in the liver as the subject scan, we use it to estimate both the background noise and the liver spillover signals in the subject scan. As means of eliminating the mass overestimation due to signal superposition from different sources, we subtract the total signal inside the CV spherical segment to the signal inside that same segment on the subject scan to approximate the signal exclusively coming from the MNPs in the ROI, and then we quantify into iron mass. In this section we present the results for CVS 100% segmentations labeled as ‘CVS’, while the results for the different percentages of $V_\mathrm{thres}$ can be seen in figure S8 of the supplementary material.

As can be seen in table S2 in the supplementary material, the brain dataset quantification was not significantly modified by using this new approach. For 2D, the overall quantification improved when compared to the threshold or CV approaches, with the relative error for the 2.5 and 1.75 $\mu$gFe test masses remaining below 10%, the 0.5 $\mu$gFe test mass reaching an exact quantification (0% relative error) and all the smaller test masses lowering the relative error (see shading in table S2 in the supplementary material). For 3D, there is an improvement in the 0.5 $\mu$gFe test mass quantification, reaching the exact quantification again but all the smaller masses slightly increasing relative error. Although this seems discouraging, the ranges given by the values and their estimation error include the ground truth mass value for all of them. For example, 0.25 $\mu$gFe was quantified as (0.27 $\pm$ 0.02) $\mu$gFe, resulting in the range [0.25, 0.29] $\mu$gFe, which we consider accurate.

**Figure 7.**
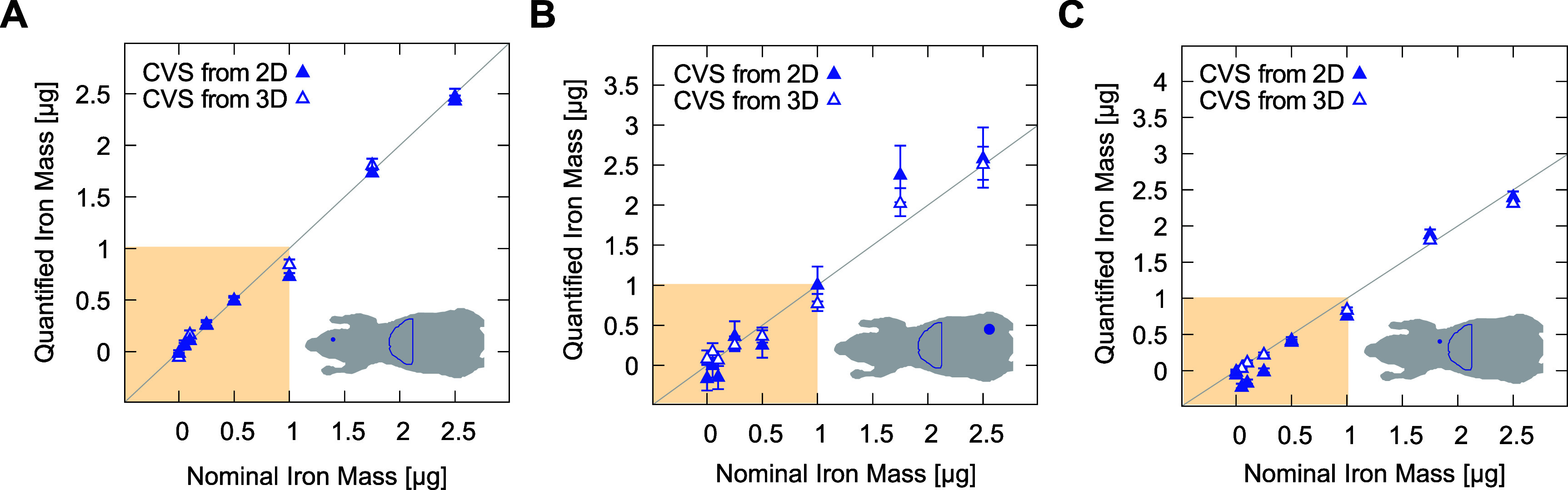
Subtraction of spillover signal enhances the limit of quantification for signals in the flank and lung of a mouse that also has strong liver accumulation. Quantified iron mass from the CVS segmentation approach as a function of nominal iron mass for the (A) brain, (B) flank and (C) lung datasets. The Threshold and CV 100% data is also presented to guide the discussion. The yellow shaded region highlights doses that represent 1%ID and lower values.

When reanalyzing the flank dataset with the CVS approach, the offset with respect to the expected-value line in the parity plots is no longer visible (see figure 7(B) for 2D). For 3D, the quantification was very slightly improved. The relative error was reduced for all the test masses except 1 and 0.5 $\mu$gFe (see shading in table S3 in the supplementary material). Thus, the CVS approach serves to subtract signal in the case of poor SNR scans. It is worth noting that the two smallest masses (0.05 and 0.1 $\mu$gFe) are still indistinguishable from 0 when considering the error, so there was no improvement in limit of quantification. This might initially sound inauspicious, but 0.1 $\mu$gFe represents 0.1%ID, substantially below the median value of 0.7%ID reaching solid tumors (Wilhelm *et al* 2016).

For the CVS analysis of the lung dataset, we see in figure 7(C) that the offsets in both the 2D and 3D CV data were removed by the subtraction. For 2D, test masses from 0.05 to 0.25 $\mu$gFe were quantified as negative or 0. For 3D, the subtraction approach produces a fair quantification for all masses, improving with respect to the one from the threshold and CV approaches, which is remarkable for a dataset with such a high level of signal spillover. While the spillover effect was strong, the SNR is high with respect to that of the flank dataset in 3D, which might be determinant for the accuracy of the quantification of low signals such as the one from test masses going from 1 to 0.05 $\mu$gFe (1%ID and below). Even more, the superposition of signal enhances the SNR in the ROI.

For easy comparison of all the quantifications presented in this article, figure S9 in the supplementary material collects the results from Threshold, CV and CVS for the three datasets.

## 4. Discussion

Due to the ethical, economic, and scientific limitations of animal testing, there is growing interest in developing faster, more cost-effective, and more informative alternatives. These alternatives, known as new approach methodologies (NAMs), are intended to complement or replace traditional animal studies. NAMs offer the potential to replace animal testing whenever feasible, reduce animal use and refine remaining *in vivo* procedures, complying with the 3Rs of animal experimentation (Bailey 2024, Sewell *et al* 2024, Rosolowski *et al* 2025). MPI is itself a NAM-friendly technique, as it allows the longitudinal imaging of subjects, reducing the number of mice used to obtain information on pharmacokinetics at different time points. For this study, we used additive manufacturing to create mouse phantoms with correct dimensions and anatomy to facilitate the controlled reproduction of biomedically relevant scenarios in terms of MNP signal intensity and separation in MPI images of mice, further adhering to the 3R’s guidelines.

The scenarios studied here are representative of systemic delivery of MNPs, characterized by dominant liver uptake and low accumulation at target sites, which is a biodistribution pattern that remains a major limitation for both quantitative imaging and therapeutic applications (Wilhelm *et al* 2016, Cheng *et al* 2020, Chen *et al* 2023). Such conditions constitute a clinically relevant case study for quantitative imaging, where doses of less than 1%ID need to be quantified in tumors, which means that there is a large dynamic range between liver and target site signal. We tackle the problem of target visualization in the presence of the liver for the first time by transforming the MPI signals logarithmically, while segmenting and quantifying in the original images with linear signal.

The brain dataset enables evaluation of internal lesions, such as intracranial tumors or hemorrhages under non-invasive imaging. The flank model represents superficial tumors of interest for magnetic hyperthermia and near-infrared radiation therapies. The lung dataset emulates small primary or metastatic tumors embedded in a challenging location, severely affected by liver spillover. Together, these clinically distinct contexts provide a systematic framework to assess the robustness, limitations, and failure modes of quantitative approaches for new imaging techniques like MPI, that show promise in terms of longitudinal assessment.

Strong off-target accumulation as in the liver can bias quantification at low-accumulation sites because of spillover effects. There are three preceding but very recent studies that report on the spillover effect in MPI images. The first one, by Sehl and coauthors (Sehl *et al* 2024), talks about it as ‘shine-through’ and identifies it as a challenge for signal separation between injection site and nearby sentinel lymph nodes in mice experiments. They used a phantom with different wells filled with MNPs separated at different distances and conclude that the shine-through resolution of MPI is 5 mm center-to-center in their imaging conditions by evaluating line scans passing through several wells. The second one, by Sanders and coauthors (Sanders *et al* 2025), reports on a physics-based reconstruction model for MPI images that improves resolution and mentions that their model could improve ‘shine-through’ resolution but do not test it. The last one, by Shakeri-Zadeh and coauthors (Shakeri-Zadeh *et al* 2025), is a comprehensive study on how the relative intensity and distance between two MPI signals affects signal quantification due to the spillover from one to the other. They evaluate the overquantification in four different scan modes and show their implications in an *in vivo* scenario, where fiducials were placed at different distances relative to the liver of the live mouse that received three consecutive injections of tracer. None of these studies attempt to quantify against the ground truth iron mass value. The paper by Sanders *et al* suggests that new reconstructions can be the way of minimizing this effect, but they do not report specific experiments to test the hypothesis.

Our study evaluates the spillover from a strong liver signal into the lower brain, flank and lung in a controlled manner, which allows us to compare with the ground truth value in the target region each time. Moreover, we evaluate the impact of the segmentation methods (threshold vs. constant volume) on the overquantification of signal and propose a strategy to subtract the spillover signal.

While only the smallest test masses in the brain (0.05 and 0.1 $\mu$gFe, representing 0.05 and 0.1%ID) pose quantification challenges with thresholded segments, more extended signals like a hind flank tumor or signals close to high accumulation sites like in the lung produce difficulties in thresholded signal delimitation that led to inaccurate quantifications or even no quantification above the 1%ID. It is however important to note that the problems are different in nature. In the flank dataset, the signal volume is more than 25 times of that of the capillary signals used for the brain and lung. By testing for the flank the same masses as in the brain and lung datasets, we end up with lower concentrations of iron that yield low signals in a larger area of the scan. First, if signals are close to noise levels, background contributes significantly to the signal. Second, when thresholding up to the 0.5$S_{\mathrm{max}}$, background can get thresholded in, especially in 2D. For the lung dataset it is evident that the proximity to the liver is playing a crucial role increasing the difficulty in thresholding signals. Mainly in 2D but also present in 3D, the threshold from the lung signal takes up spurious signal that is decaying from the liver (i.e. liver spillover), which is evident when looking at the shape of the thresholded segments (figure 3(E), 2.5 $\mu$gFe from 2D volumes and 0.05 $\mu$gFe from 3D volumes), which are joining of the lung capillary signal and a ring around the liver signal.

When evaluating constant volume segments, we obtained an offset in the parity plots for the flank signal quantification in 2D. We proposed that because of poor SNR, the background levels were added to the MNP signal and both contributed significantly to the total signal measured. The strongest evidence supporting this conclusion is that the effect disappears when the signal is quantified in 3D, where the SNR is higher. For the lung, although the ROI signal values are higher than in the flank (see figures 2(B) and (C)), this offset is also observed in the quantification of high-SNR 3D images. This hints that background levels are improbable to be the source of the offset in the lung’s case and that the liver spillover evidenced through the thresholded shapes is in this case the cause of the offset.

The problems in the quantification of the flank and lung dataset have a different origin but manifest as the same (i.e. overestimation of the mass). Whether the additional contribution to the signal is noise (flank in 2D) or liver spillover (lung both in 2D and 3D), the background subtraction improved quantification. This background subtraction accounted for liver signal present when studying the target regions through the acquisition of a reference scan that encompassed just the liver signal.

The study of the spillover effect in controlled scenarios where we can access ground truth iron mass values advances the interpretation of tracer accumulation data. *In vivo*, off-target accumulation in organs such as the liver/spleen, can obscure or distort quantification of weaker signals from target tissues such as tumors, known to attain low accumulation from systemic delivery. There is the need to think about how to translate or adapt the subtraction approach used here to quantification of images from living mice and eventually humans. While the threshold and CV segmentations are easily translated to the analysis of *in vivo* data, the subtraction approach is more difficult to implement and would involve the acquisition of a representative reference scan for the application being studied. Shakeri-Zadeh *et al* (2025) showed spillover *in vivo* when the MNPs have already accumulated in the liver, but tracers have different half-lives so reference scans will vary by tracer and timepoint. As MPI allows one to perform longitudinal studies, one possibility would be obtaining both early-timepoint kinetics and long-term accumulation data on the same subject and utilizing the latter to subtract from the early timepoints. Another possibility would be characterizing a typical liver/spleen signal distribution and predicting signal decay from the off-target site into the ROI, which could be done with machine learning methods or in a more traditional way but would require the acquisition of many images of liver/spleen accumulation with different tracers and different subjects.

Recently, the first MPI scans in human volunteers were reported in preprints, one of them focusing on single timepoint angiography (Vogel *et al* 2026) and another one showing two longitudinal and quantitative studies of particle accumulation and clearance from the scalp and foot (Mason *et al* 2026). This landscape should motivate the MPI research community to develop solid methodologies guiding biomedical translation more than ever.

## 5. Conclusions

Reliable MPI signal quantification *in vivo* requires the systematic evaluation of segmentation strategies, for which anatomically accurate phantoms serve as a powerful platform that minimizes animal use in research. In this work, we employed a 3D-printed, anatomically correct mouse phantom to systematically evaluate quantification strategies in MPI applications, mimicking tumor and long-term liver nanoparticle accumulation. The relative distance of the signals with respect to the liver allowed us to evaluate spillover, which is a common challenge *in vivo*.

We explored the quantification accuracy for several segmentation-based approaches in brain, lung and flank regions in the presence of the liver signal. The results showed that for isolated, highly concentrated signal, simple threshold-based methods provide reliable quantification even below the 1% of the ID mark. However, in regions close to high-signal organs (e.g. the lung adjacent to the liver) or regions with low nanoparticle concentration (e.g. the flank), thresholding was prone to overestimation due to spillover or poor SNR ratio effects. The use of constant-volume segmentations allowed to separate signals that were shadowed by background noise or other organs, while the subtraction strategies proved beneficial in mitigating the overestimation due to spillover signal and improving the limit of quantification, which is particularly important for the design and outcome understanding of nanoparticle-based treatments.

Overall, our study highlights the need of accounting for superimposed signals when interpreting *in vivo* data quantitatively, and how the use of anatomically-correct phantoms is essential to test new quantification strategies in biologically-relevant scenarios to help the translation of preclinical findings to animals and, ultimately, humans.

## Data Availability

The data that support the findings of this study are openly available at the following URL/DOI: https://doi.org/10.5061/dryad.59zw3r2pz (Valdés 2026). The models for the bed and phantom used in this study can be found in our GitHub repository. Supplementary data available at: https://doi.org/10.1088/1361-6560/ae91c7/data1.
